# How physical activity protects against smartphone addiction: examining the mediating pathways of resilience and subjective wellbeing in Chinese university students

**DOI:** 10.3389/fpsyg.2025.1704827

**Published:** 2026-01-09

**Authors:** Huiyu Shi, Yuan Zhang, Zhihui Li, Jian Yang

**Affiliations:** 1 College of Physical Education and Health, East China Normal University, Shanghai, China; 2 College of Physical Education and Health, Hunan University of Technology and Business, Changsha, Hunan, China

**Keywords:** China, phone addiction, physical activity, resilience, subjective wellbeing, university students

## Abstract

**Background:**

Phone addiction has become a global concern, and empirical evidence indicates that physical activity is negatively associated with phone addiction. However, the mediating roles of resilience and subjective wellbeing in this relationshihp have scarcely been studied, especially in Chinese university students. The present study aimed to explore the direct and indirect associations between physical activity and phone addiction through the mediating roles of resilience and subjective wellbeing in Chinese university students.

**Methods:**

This cross-sectional study recruited 515 Chinese university students. Physical activity, resilience, subjective wellbeing, and phone addiction were assessed using validated self-report questionnaires. Pearson’s correlations were conducted to examine associations among variables, and mediation analyses were performed using bias-corrected bootstrap procedures in AMOS 26.0 and RStudio.

**Results:**

Of the four variables that showed significant correlations, the association between physical activity and lower levels of phone addiction was specifically mediated only by subjective wellbeing and by the chain pathway involving both resilience and subjective wellbeing.

**Conclusion:**

Within the specific context of educational involution in China, this study provides a specific insight into the relationship between physical activity and phone addiction. Promoting physical activity may be beneficial as a preventive strategy to address phone addiction. Further research is required to expand these results in diverse populations.

## 1 Introduction

In the cyber age, smartphone has become ubiquitous in daily life, based on a global estimated 6.7 billion smartphone subscriptions, and are expected to continuously increase in the following 5 years (Statista, 2024). Smartphone, providing interaction, entertainment and information platform (Mei et al., 2023) without time and geographical restrictions, has highly attracted university students as a multitool. However, benefits of smartphone may enhance risks of excessive use, which has become a worldwide health concern proposed by the World Health Organization (WHO) (2014). With higher levels of digital competence and literacy, university students are more likely to abuse smartphones. In a meta-analytic review, it is estimated that the global pooled prevalence of smartphone addiction was 26.96% among university students (Meng et al., 2022), which lead to a series of problematic behaviors including distraction in class (Junco, 2012), academic procrastination (Aznar-Díaz et al., 2020), depression (Geng et al., 2021), anxiety, sleep problems (Demirci et al., 2015), and physical problems (Mei et al., 2023). Terms coined by different scholars including “Problematic Mobile Phone Use” (PMPU), “Phone Addiction,” “Phone Dependence” and “Phubbing” refer to the behavior that overuse phones. Billieux et al. (2015), who figured out a comprehensive model for disordered mobile phone use, divides problematic phone use into three types: (a) addictive pattern (i.e., addiction symptoms and reassurance behaviors), (b) antisocial pattern (i.e., prohibited use, inappropriate use in specific contexts and phone aggression) and (c) risky pattern (i.e., calling while driving and dangerous texting). Although the latest edition of the Diagnostic and Statistical Manual of Mental Disorders (DSM-5; APA, 2022) and International Classification of Diseased (ICD-11; World Health Organization, 2021a) do not list phone use problems as a specifically addictive disorder, Bianchi and Phillips (2005) argued that addictive pattern of phone use is a behavioral addiction, manifested by the presence of tolerance, escaping, withdrawal, craving, and negative life consequences. In brief, it is quite important to delve into phone addiction given its harmful consequences.

Physical inactivity has also become an increasingly serious problem worldwide, which cause 6–10% of the major non-communicable diseases of coronary heart disease, type 2 diabetes, and breast and colon cancers (Lee et al., 2012). It is estimated that in 2022, the prevalence of insufficient physical activity was nearly a third of global adults (31.3%, 1.8 billion; Strain et al., 2024) and the World Health Organization (2021b) recommended that all adults should perform at least 150 to 300 min of moderate aerobic activity per week (or the equivalent vigorous activity). Apart from considerable physical health benefits, convergent evidence indicate the indispensable role of physical activity in primary prevention and clinical treatment across a spectrum of mental disorders including depression and anxiety (Firth et al., 2020).

### 1.1 The relationship between physical activity and phone addiction

Accordingly, physical activity tends to promote positive health behavior changes (Hall et al., 2015). Given that many findings propose the correlation between physical activity and phone addiction (Lu et al., 2022), physical activity has been regarded as a potential therapeutic modality to reduce the level of phone addiction (Awick et al., 2017). In other words, physical activity is a protective factor of phone addiction. Previous studies have suggested that physical activity can have a negative association with phone addiction by reducing negative emotions among university students (Tong et al., 2023; Ke et al., 2024; Wang et al., 2024, 2025). However, the pathway between these two variables is unclear till now. Therefore, it is postulated that:

*H1*: Physical activity is negatively associated with phone addiction.

### 1.2 The mediating role of resilience

Resilience, a vital source of mental health, embodies a personality or trait that enable one to thrive in the face of adversity (Connor and Davidson, 2003). It is also defined as a dynamic process in which individuals adapt to significant adversity or trauma positively (Luthar and Cicchetti, 2000). Physical activity has been shown to have positive effects on resilience, and the mechanisms whereby physical activity promotes resilience are diverse (Silverman and Deuster, 2014). Physical activity may confer resilience via serving as a buffer against stress and stress-related disorders according to the theory of cross-stressor adaption (Sothmann et al., 1996). Based on cognitive-affective neuroscience framework, Belcher et al. (2021) argue that physical activity may act as resilience factors by promoting structure and function of neural circuits involved in self-regulation. In a cross-sectional study, it has been found that people who exercise regularly perform higher level of resilience, and thus physical activity could positively predict resilience (Yoshikawa et al., 2016). Moreover, resilience proves to negatively affect phone addiction (Ma et al., 2022), which indicates those with higher level of resilience are less likely to overuse smartphone. One study indicated that a dynamic relationship exists between resilience and phone addiction, in which resilience has a negative effect on the level of phone addiction after 6 months (Dong et al., 2023). Resilient individuals, who have more internal resources to deal with stress and adversity, tend to obsess less with internet use to control their emotions (Nam et al., 2018). Thus, it is postulated that:

*H2*: Resilience mediates the association between physical activity and phone addiction.

*H2a*: Physical activity is positively related to resilience.

*H2b*: Resilience is negatively associated with phone addiction.

### 1.3 The mediating role of subjective wellbeing

Subjective wellbeing, in the area of positive psychology, is used to describe people’s cognitive and affective evaluations of their lives, including life satisfaction, positive affect and negative affect (Diener, 2000). A growing body of evidence suggest that physical activity makes a significant contribution to positive mental health and wellbeing via neurobiological, psychosocial, and behavioral mechanisms (Lubans et al., 2016; Kandola et al., 2019; Roychowdhury, 2020). A study of the university population suggests that these students tend to have stronger subjective wellbeing when they participate in sport activities (Brand et al., 2020). Graupensperger et al. (2020) also remark that physical activity can offer more social support and can effectively promote individuals’ mental health and subjective wellbeing. In addition, some findings indicate a positive relationship between subjective wellbeing and phone owing to its social use (Chen and Li, 2017), while more studies suggest that lower wellbeing is correlated with phone addiction (Kumcagiz and Gunduz, 2016; Wang et al., 2024a). Based on the model of compensatory internet use (Kardefelt-Winther, 2014), university students with lower subjective wellbeing have worse adaption to surroundings and thus they might seek for the help of phone (Chen, 2017). Phone addiction may exist, if the positive stimuli it provides constantly compensate for negative affective brought from the ideal-reality gap. Accordingly, it is postulated that:

*H3*: The association between physical activity and phone addiction is mediated by subjective wellbeing.

*H3a*: Physical activity is positively related to subjective wellbeing.

*H3b*: Subjective wellbeing is negatively associated with phone addiction.

### 1.4 The chain mediation of resilience and subjective wellbeing

To date, bulk of studies have found a correlation between resilience and subjective wellbeing (Lü et al., 2014; Doyle et al., 2016). Top-down theories proposed by Diener and Ryan (2009) claim that traits are important predictors of subjective wellbeing. Compared to a person with a more negative perspective, a person with a more positive state of mind may interpret the objective events as “happier.” Therefore, resilience, as a process or a trait of positively bouncing back from adversity, has an overall impact on individual’s subjective wellbeing. Some scholars have discovered that resilience can be regarded as a human strength to enhance subjective wellbeing (Doyle et al., 2016). Similarly, Satici (2016) suggests that higher levels of resilience may provide university students with hope, which helps them evaluate the cognitive and affective dimensions of life more positively. Hence, it is postulated that:

*H4*: Resilience and subjective wellbeing are chain mediators of the association between physical activity and phone addiction.

*H4a*: Resilience is positively correlated with subjective wellbeing.

In brief, the present study aims to reveal the direct and indirect correlations between physical activity and phone addiction among Chinese university students, via the mediating role of resilience and subjective wellbeing. Specifically, we hypothesize that physical activity is negatively associated with phone addiction (H1). We further propose that resilience mediates this relationship (H2), and that subjective wellbeing also serves as a mediator between physical activity and phone addiction (H3). In addition, given evidence showing associations between resilience and subjective wellbeing, we hypothesize a chain mediation model in which these two variables are sequentially linked in the relationship between physical activity and phone addiction (H4).

## 2 Materials and methods

### 2.1 Participants and procedure

The present study employed a cross-sectional design and surveyed a convenience sample of participants in China. Prior to data collection, Monte Carlo simulations were conducted to estimate the sample size needed to achieve adequate statistical power for detecting the hypothesized mediation and serial mediation pathways. Assuming standardized medium effect sizes and a standard deviation of 1 for all variables, sample sizes ranging from 50 to 1,000 were tested with 5,000 replications per condition. The results indicated that the power for detecting single indirect effects increased from 0.15–0.23 at *N* = 50 to 0.99 at *N* = 500, and the full serial mediation pathway reached virtually 1.00 power at *N* = 500. Based on these simulations, an effective sample size of approximately 500 participants was targeted to ensure valid and reliable statistical power. A total of 591 questionnaires were collected both on campus using paper-based materials (flyers containing QR codes) and through online platforms such as WeChat and QQ. The questionnaire was administered electronically via Questionnaire Star to facilitate convenient and efficient data collection. All participants were at least 18 years old. The demographic characteristics of the sample are presented in Table 1. In accordance with the ethical review system and the approval by the Ethics Committee of East China Normal University (HR 512–2024), participants over 18 years of age were not required to sign informed consent forms. All participants were fully informed about the purpose and content of the study, and were reminded that the survey was anonymous, voluntary, and that they could withdraw at any time. To ensure data quality, invalid questionnaires were excluded based on the following criteria: (1) participants failed the attention check item (“If you are completing this questionnaire carefully, please choose ‘1’”); (2) participants gave the same response to all reverse-coded items. After applying these criteria, 515 valid questionnaires remained for analysis.

**Table 1:** Mean, standard deviation, distribution parameters and internal consistency of study variables.

Variables	Ratio	*M* (SD)	*α*
Gender %			
Male	44.466		–
Female	55.534		–
Major %			
Sports-related	35.534		–
Others	73.204		–
Education level %			
Bachelor	94.951		–
Master	4.466		–
PhD	0.583		–
Type of physical activity %			
Team sports	38.252		–
Individual sports	61.748		–
Age *M* (SD)		20.181 (1.768)	–
Physical activity *M* (SD)		29.041 (28.861)	0.802
Phone addiction *M* (SD)		51.748 (11.843)	0.874
Resilience *M* (SD)		27.140 (7.211)	0.946
Subjective wellbeing *M* (SD)		10.452 (1.802)	0.874

### 2.2 Measures

The online survey comprised the following: (a) demographics, (b) Physical Activity Rating Scale (PARS-3), (c) Mobile Phone Addiction Index (MPAI) Scale, (d) Connor-Davidson Resilience (CD-RISE) Scale, and (e) Index of Well-Being and Index of General Affect (IWB and IGA) Scale. Specific item was designed to exclude invalid questionnaires (e.g., “If you are doing this questionnaire carefully, please choose ‘1″’).

The demographics examined included gender, age, major (i.e., sports-related major and others), education level (i.e., Bachelor, Master and PhD), and main type of physical activity (i.e., team sports and individual sports).

PARS-3 (Liang, 1994): The PARS-3 was chosen to assess physical activity. The three items comprised: (a) intensity (How would you describe the intensity of the physical activity you have participated in on average?) on a 5-point scale from 1 to 5 points; (b) duration (How long have you participated in the above physical activity each time on average?) on a 5-point scale from 0 to 4 points; and (c) frequency (How often have you been involved in the above physical activity?) on a 5-point scale ranging from 1 to 5 points. Total scores categorize activity as light (0–19), moderate (20–42), or vigorous (43–100). The PARS-3 does not differentiate between dimensions and thus participants also reported whether their activity was mainly team-based or individual.

MPAI Scale (Leung, 2008; Huang et al., 2014): The 17-item MPAI Scale was employed to assess phone addiction ranging on a 5-point scale from 1 to 5 points (1 = not at all; and 5 = always). The four subscales are: (a) Inability to Control Craving (e.g., Your friends and family complained about your use of the mobile phone), (b) Feeling Anxious & Lost (e.g., When out of range for some time, you become preoccupied with the thought of missing a call), (c) Withdrawal/Escape (e.g., You have used your mobile phone to talk to others when you are feeling isolated), and (d) Productivity Loss (e.g., You find yourself occupied on your mobile phone when you should be doing other things, and it causes problem). The total scores ranged from 0 to 85, with higher scores representing a higher likelihood of problematic smartphone use.

CD-RISC Scale (Campbell-Sills et al., 2007; Wang et al., 2010): 10-item CD-RISC Scale was used to assess resilience (e.g., Able to adapt to change) ranging on a 5-point scale from 0 to 4 (0 = not true at all; 4 = true nearly all the time). The total scores ranged from 0 to 40, with higher scores representing higher levels of resilience.

IWB and IGA Scale (Campbell, 1976): 8-item IWB and 1-item IGA Scale were used to assess subjective wellbeing, ranging on a 7-point scale from 1 to 7 points (i.e., 1 is assigned to the least favorable response, 7 is assigned to the most favorable, and 2–6 is assigned to intervening responses). The IWB subscale comprises eight items (e.g., 1 = boring, 7 = interesting). The IGA Scale contains one item (“How satisfied are you with your life as a whole these days?”) and was scored from 1 to 7 (1 = Unsatisfied, 7 = Satisfied). The total scores were evaluated by the sum of the IWB (*1.0) and IGA (*1.1).

### 2.3 Statistical analysis

To minimize common method bias, some questionnaire items were reverse-coded, responses were collected anonymously, and Harman’s (1976) one-way validation method was applied. A descriptive study with internal consistency (Cronbach’s *α*; Hayes and Coutts, 2020) was conducted. Given the large sample size and acceptable skewness and kurtosis values (|skewness| < 2, |kurtosis| < 7), the data were considered approximately normal (West et al., 1995). Pearson’s correlation coefficient was used to identify the relationships between the variables. Subsequently, bias-corrected bootstrapped estimates (Efron and Tibshirani, 1994), based on 5,000 bootstrapped samples, were used to identify the association between physical activity (predictor), resilience (mediating variable) and subjective wellbeing (mediating variable) on problematic smartphone use (dependent variable). Covariates were included in the models to control for potential confounding effects. Exploratory analyses were also conducted including multi-group comparisons to assess potential differences across subgroups. Data analyses and visualizations were performed using Amos 26.0 and RStudio (v. 2024.04.2, R environment v. 4.4.1) using the GGally and ggplot2 packages.

## 3 Results

### 3.1 Sample demographics

Harman’s one-way validation indicated that eight factors with eigenvalues greater than 1 were extracted, and the maximum factor variance explained was 21.915%, which is below the 40% threshold, suggesting that common method bias was not a serious concern. Table 1 shows the descriptive statistics of the study variables in the Chinese samples as well as the internal consistencies of each measure.

### 3.2 Correlation

**Figure 1:**
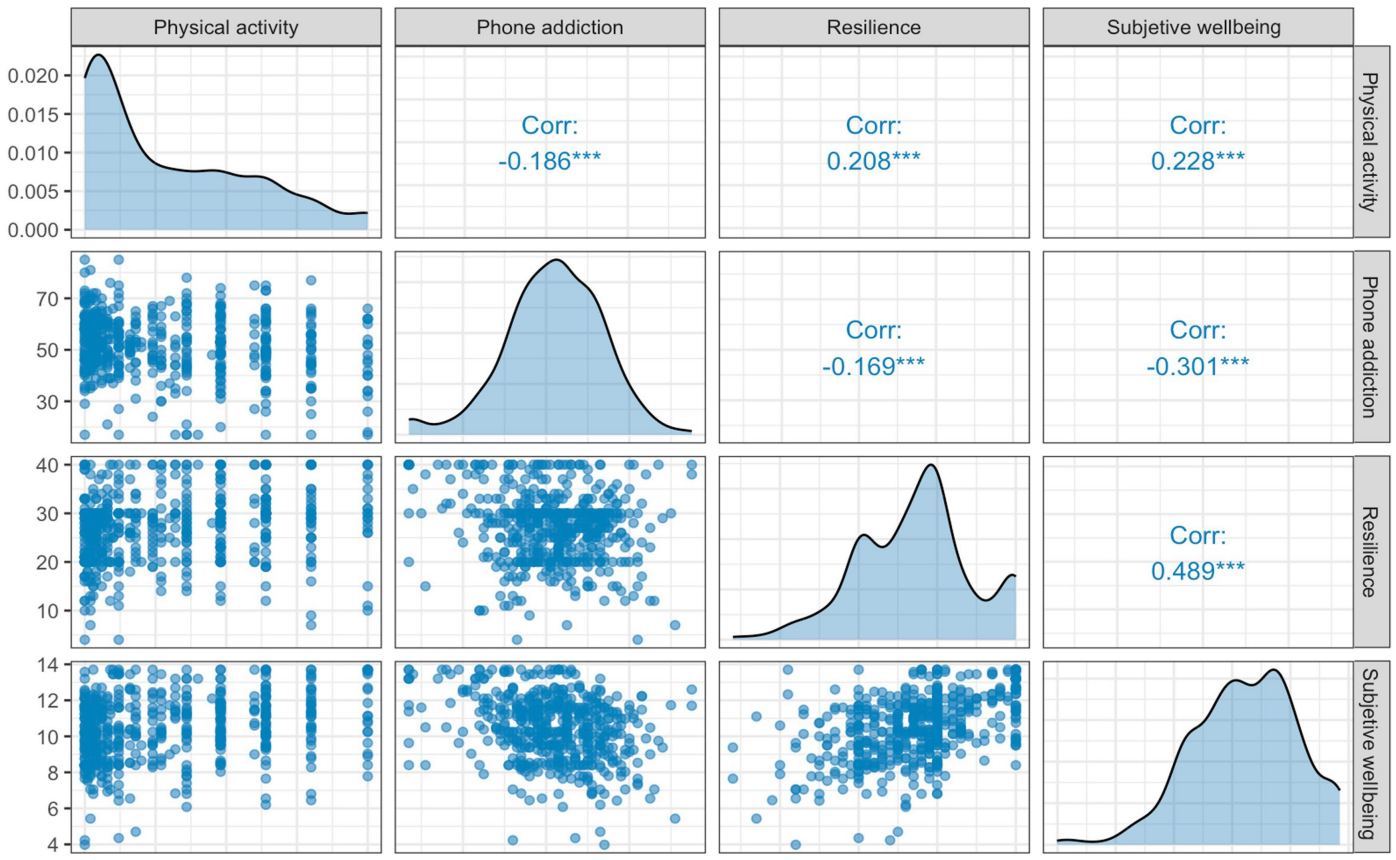
Correlation matrix of the main variables. The figure displays scatterplots showing the distribution of values for each pair of variables, density plots of standardized scores (*z*-scores) along the diagonal, and the strength of correlations between variables. Numbers in the matrix represent Pearson’s correlation coefficients (*r*). ***p < 0.001.

The correlations between the main variables across the dataset are shown in Figure 1. Physical activity was positively correlated with resilience (*r* = 0.208, *p* < 0.001) and subjective wellbeing (*r* = 0.228, *p* < 0.001). Similarly, resilience was positively correlated to subjective wellbeing (*r* = 0.489, *p* < 0.001). Phone addiction was negatively correlated with physical activity (*r* = −0.186, *p* < 0.001) and subjective wellbeing (*r* = −0.301, *p* < 0.001). A significant correlation between resilience and phone addiction could be observed (*r* = −0.169, *p* < 0.001).

### 3.3 Mediational model

Subsequently, we examined the associations between physical activity with phone addiction, as well as the mediating role of resilience and subjective wellbeing in this relationship. The results of the model fit are as achieved: x^2^ /df = 2.537, GFI = 0.981, TLI = 0.965, CFI = 0.980, and RMSEA = 0.055. As shown in Table 2, the direct pathway between physical activity and phone addiction had a standardized path coefficient of −0.118 (*95%CI:* −0.217, −0.019), supporting Hypothesis 1. Physical activity was positively associated with resilience [*β* = 0.208, (*95%CI:* 0.115, 0.296)], supporting Hypothesis 2a, whereas resilience was not significantly associated with phone addiction [*β* = 0.052, (*95%CI:* −0.083, 0.185)], against Hypothesis 2b. Physical activity was positively related to subjective wellbeing [*β* = 0.179, (*95%CI:* 0.093, 0.264)], supporting Hypothesis 3a, and subjective wellbeing was negatively related to phone addiction [*β* = −0.388, (*95%CI:* −0.524, −0.244)], supporting Hypothesis 3b. Resilience was positively associated with subjective wellbeing [*β* = 0.540, (*95%CI:* 0.448, 0.615)], supporting Hypothesis 4a (See in Table 2).

**Table 2:** Summary of the direct effect of the model paths.

Path	*β*	95%*CI*
Physical activity → Phone addiction	−0.118	(−0.217, −0.019)
Physical activity → Resilience	0.208	(0.115, 0.296)
Resilience → Phone addiction	0.052	(−0.083, 0.185)
Physical activity → Subjective wellbeing	0.179	(0.093, 0.264)
Subjective wellbeing → Phone addiction	−0.388	(−0.524, −0.244)
Resilience → Subjective wellbeing	0.540	(0.448, 0.615)

Standardized path coefficients (*β*) for direct associations among variables. Significance was determined using 95% bias-corrected bootstrapped confidence intervals (5,000 samples) that did not include zero.

**Figure 2:**
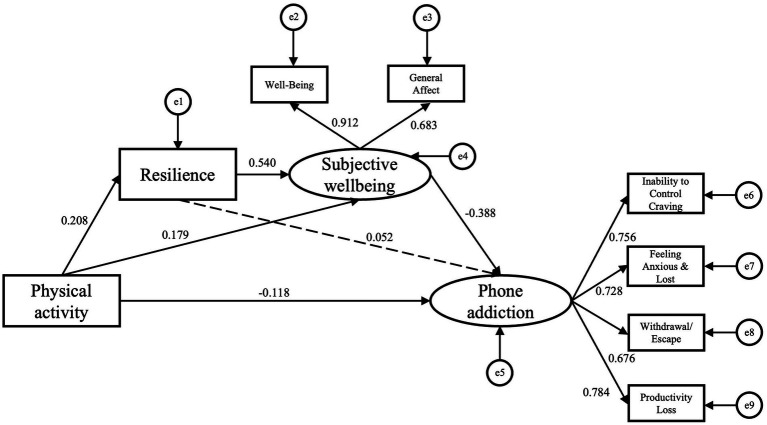
Structural equation model showing standardized path coefficients (*β*) for associations between physical activity (independent variable) and phone addiction (dependent variable), with resilience and subjective wellbeing as mediators. Solid lines indicate significant paths, while dotted lines represent nonsignificant paths. Numbers on the paths correspond to standardized path coefficients (*β*) determined using 95% bias-corrected bootstrapped confidence intervals (based on 5,000 samples).

The indirect pathways of the structural equation model can be seen in Table 3. The total associations of physical activity, resilience, and subjective wellbeing with phone addiction were significant. Specifically, the indirect pathway between physical activity and phone addiction contained 2: (a) subjective wellbeing mediated the association between physical activity and phone addiction [*β* = −0.069, (*95%CI*: −0.116, −0.034)], accounting for 33.173% of the total effects, supporting Hypothesis 3. (b) Resilience and subjective wellbeing sequentially mediated the association between physical activity and phone addiction [*β* = −0.044, (*95%CI*: −0.076, −0.021)], representing 21.153% of the total effects, supporting Hypothesis 4. Against Hypothesis 2, resilience did not mediate the association between physical activity and phone addiction [*β* = 0.011, (*95%CI*: −0.016, 0.041)]. The mediational model is shown in Figure 2.

**Table 3:** Summary of the direct and indirect effects of model paths.

Path	*β*	95%*CI*
Total effect	−0.219	(−0.314, −0.123)
Direct effect	−0.118	(−0.217, −0.019)
Physical activity → Phone addiction		
Pathway 1	0.011	(−0.016, 0.041)
Physical activity → Resilience → Phone addiction		
Pathway 2	−0.069	(−0.116, −0.034)
Physical activity → Subjective wellbeing → Phone addiction		
Pathway 3	−0.044	(−0.076, −0.021)
Physical activity → Resilience → Subjective wellbeing → Phone addiction		

Standardized path coefficients (*β*) for indirect and total effects among variables. Significance was determined using 95% bias-corrected bootstrapped confidence intervals (5,000 samples) that did not include zero.

Exploratory multi-group SEM invariance tests were conducted to examine whether the type of physical activity (team vs. individual) moderated the hypothesized model. The unconstrained and constrained models demonstrated good fit (CFI > 0.97, TLI > 0.97, RMSEA < 0.05). Changes in fit indices between models were minimal up to the structural covariance level (ΔCFI ≤ 0.01), indicating that both measurement and structural models were invariant across groups (Cheung and Rensvold, 2002). These results suggest that the structural relationships were largely equivalent across team- and individual-sports groups, highlighting the stability of the model across groups (see Supplementary materials). Sensitivity analyses also using different numbers of bootstrap samples (2,000 and 10,000) confirmed that the parameter estimates and indirect effects were robust across analytical conditions.

## 4 Discussion

The present study aimed to investigate the direct and indirect associations between physical activity and phone addiction through the mediating role of resilience and subjective wellbeing in Chinese university students. These findings partially supported our hypotheses.

### 4.1 Physical activity and its negative association with phone addiction

Physical activity was significantly associated with phone addiction, indicating that those with inactive physical activity levels were more likely to be addicted to their phones. This result was consistent with previous findings in the introduction (Tong et al., 2023), and supported Hypothesis 1. One possible reason for this is that physical activity may take up the time that individuals spend on smartphone (Abbasi et al., 2021), which relieves phone addiction. On the other hand, from the perspective of neurobiological mechanisms, physical activity can regulate neurotrophic factor, secrete anti-inflammatory factors, promote nerve growth and differentiation, improve brain plasticity, and remodel dopamine reward circuits to lower the possibility of phone addictive behavior (Pedersen and Febbraio, 2008; Kandola et al., 2019). Moreover, phone addiction is often linked to mental health (Geng et al., 2021), while physical activity may also bring obvious psychological benefits to individuals (Firth et al., 2020). In the last, differences in the type of physical activity might trigger different effects on phone addiction (Yang et al., 2021), in which team sports with competitive, playful, and social characteristics, such as basketball, present higher affect phone addictive behavior (Xiao et al., 2021). More importantly, maintaining face-to-face communication with teammates and coaches while doing exercise, as well as being immersed in the joy of sports, might satisfy university students’ needs for social interaction and entertainment rather than depending on smartphone applications.

### 4.2 Reevaluating the role of resilience

Surprisingly, regarding the role of resilience in the association between physical activity and phone addiction, the results partially rejected the Hypothesis 2. Physical activity was positively associated with resilience, which verified Hypothesis 2a and was in line with previous findings (Yoshikawa et al., 2016). Under the cognitive-affective neuroscience framework, physical activity confers resilience through top-down control of bottom-up processing to strengthen self-regulation, which facilitates problem-solving skills (Belcher et al., 2021). Those who usually participate in physical activity are more likely to be exposed to challenges, injury, and failure, thus enhancing their resilience. However, resilience was not insignificantly associated with phone addiction when all the variables were included, thus rejecting Hypothesis 2b. Individuals with high levels of resilience were still likely to be addicted to phones, which was inconsistent with previous studies (Nam et al., 2018; Ma et al., 2022). Interestingly, this inconsistency echoes Shen (2020), who found that psychological resilience was not directly associated with excessive phone use, but moderated the association between motivational factors (e.g., escapism and social interaction) and phone use. This suggests that resilience may primarily exert its protective effect in interaction with other psychological factors, rather than functioning independently. When phone use is primarily driven by escapism motivation, individuals seek to alleviate negative affect through technological means. Returning to the definition of resilience mentioned in the introduction, using phones could serve as a coping strategy in the recovery process, albeit a negative one. According to the self-medication hypothesis, which can also be applied to technological uses, individuals use substances to regulate emotions because of insufficient coping skills (Khantzian and Khantzian, 1997). Even individuals with high resilience may struggle to distinguish whether they adapt to trauma via external (e.g., digital) or internal psychological resources, and might still turn to phone as a convenient tool for emotion regulation, particularly when cognitive resources and positive affect are not fully mobilized. Therefore, the relationship between resilience and phone addiction is likely contingent on additional psychological or contextual factors, which helps explain why resilience alone did not show a significant direct effect in our chain mediation model.

### 4.3 The mediating role of subjective wellbeing

This study found a significant mediating role of subjective wellbeing in the association between physical activity and phone addiction, which was in favor of the Hypothesis 3. Physical activity positively predicted subjective wellbeing, which supported previous studies (Brand et al., 2020), thus verifying Hypothesis 3a. Entertainment and satisfaction stemming from physical activity may increase individuals’ subjective wellbeing. Both acute and chronic effects of exercise may contribute to subjective wellbeing. On the one hand, owing to the release of endorphins in the process of acute exercise, it might largely ease pain and produce a feeling of euphoria (Dishman and O’Connor, 2009), leading to an increase in subjective wellbeing. On the other hand, the majority of previous studies on the chronic psychological benefits of physical activity confirm that regular physical activity might improve subjective wellbeing (Wicker and Frick, 2016). The results also indicated that subjective wellbeing was negatively related to phone addiction, which was in favor of previous findings (Kumcagiz and Gunduz, 2016), supporting Hypothesis 3b. Focusing on the model of compensatory internet use (Kardefelt-Winther, 2014), individuals with negative emotions or lower wellbeing preferred online activities to compensate for psychosocial problems, which may result in the possibility of phone addiction to escape problems and challenges in the real world. Accordingly, it was not surprisingly to find that subjective wellbeing partially mediated inactive physical activity and phone addiction (Lu et al., 2022). It is argued that physical activity might trigger the pituitary gland to secrete endorphins, by competing with addictive substances in the central nervous system for receptors and induce happiness, thus suppressing addiction (Jović et al., 2011). Therefore, positive emotion from acute and regular physical activity encourages individuals to get away from phone, alleviating phone addiction.

### 4.4 The chain mediating role of resilience and subjective wellbeing

Another pathway between physical activity and phone addiction was the chain mediating role of resilience and subjective wellbeing, which supports Hypothesis 4. The significant correlation between resilience and subjective wellbeing confirmed the findings of previous studies (Lü et al., 2014; Doyle et al., 2016), supporting Hypothesis 4a. Resilient individuals do not easily dwell on the negative emotions from trauma, and thus they tend to be entitled with higher level of positive affective (Satici, 2016). In addition, university students with higher resilience and subjective wellbeing showed a stronger association between physical activity and phone addiction. The observed chain mediating role of resilience and subjective wellbeing is consistent with theoretical frameworks. Under the Interaction of Person-Affect-Cognition-Execution (I-PACE) model (Brand et al., 2016), problematic internet use arises from the interaction of predisposing personal characteristics, affective and cognitive responses to internal or external stimuli, and executive functioning. In this conceptualization, resilience functions as a predisposing personal resource that shapes how individuals appraise and respond to stressors (cognitive responses) and how they regulate emotions (affective responses). Crucially, resilience alone does not directly dictate maladaptive behavioral outcomes; instead, its effect manifests through proximal affective–cognitive states that more directly govern behavior. Consistent with this model, subjective wellbeing, encompassing both positive affect and cognitive evaluations of life satisfaction, captures these proximal states. When subjective wellbeing is included in the model, the direct association between resilience and phone addiction diminishes, suggesting that resilience exerts its influence primarily through this affective–cognitive pathway. In other words, resilient individuals tend to experience higher subjective wellbeing, which in turn reduces the likelihood of engaging in problematic phone use. Resilience therefore mitigates the tendency toward maladaptive behaviors predominantly when it translates into enhanced positive affect and adaptive cognitive appraisals—the mechanisms that reduce reliance on compensatory coping strategies. The self-medication perspective further clarifies why resilience alone may be insufficient in some cases. As discussed earlier, individuals often resort to technological use as an emotion-regulation strategy when coping resources are strained (Khantzian and Khantzian, 1997). Even those with high resilience may temporarily rely on accessible means, such as smartphone use, to manage stress if their affective state or cognitive resources are depleted. Consequently, resilience must foster corresponding increases in positive affect and cognitive resources—reflected in higher subjective wellbeing—to effectively diminish the motivation for phone use as an emotional coping mechanism. This reasoning aligns with our empirical finding that resilience’s protective effect against phone addiction operates indirectly, through enhanced subjective wellbeing, rather than building a direct behavioral association.

### 4.5 The context of Chinese educational involution

The specific context of educational involution in China (Yang et al., 2023) may further intensify this process. Under sustained high pressure, students face frequent demands on attention and self-control, making them more inclined to seek rapid emotional relief through easily accessible means such as phone use. In such an environment, the immediate rewarding effects of phone engagement become particularly salient. Therefore, resilience must translate into real, momentary increases in positive affect and cognitive resources to offset these strong, contextually driven motivations for phone use. Physical activity may be especially effective in this regard, as it not only strengthens resilience through repeated exposure to challenges and failures but also enhances subjective wellbeing by promoting positive affect and constructive cognitive appraisal. In this sense, physical activity addresses both the predisposing personal traits and the proximal affective–cognitive mechanisms emphasized by the I-PACE model. Consequently, within the Chinese educational involution context, the chain pathway through subjective wellbeing becomes especially prominent, helping explain why resilience alone did not exhibit a significant direct association with phone addiction in our sample.

### 4.6 Limitations and implications

The present study had several limitations. First, the data were collected using a cross-sectional correlational design, which limits causal inference; therefore, the observed associations between physical activity, resilience, subjective wellbeing, and phone addiction should not be interpreted as causal. A longitudinal or experimental design is recommended for future studies to further examine potential causal relationships. Second, although Harman’s single-factor test suggested that common method bias was not a serious concern, the exclusive reliance on self-report questionnaires may still be subject to social desirability, response style, and memory biases, which could influence the observed associations among variables. Future studies could combine self-reports with objective measures, such as heart rate monitoring or functional near-infrared spectroscopy, to reduce potential common method bias and comprehensively investigate the relationships between physical activity and phone addiction. Third, the convenience sample of university students and the limited sample size may constrain the generalizability of the results. In addition, the relatively small sample limits the ability to examine potential dose–response relationships between physical activity levels and phone addiction. Thus, it is proposed that future research validate the findings using larger and more diverse samples, which would not only enhance generalizability but also enable stratified or dose–response analyses. Although this study was conducted exclusively among Chinese university students, future work should explore whether these findings extend to other demographic groups and cultural contexts. Fourth, the present study used the PARS-3 to assess overall physical activity. While the brevity of the scale facilitated data collection, it does not differentiate between critical dimensions such as aerobic versus anaerobic activity or habitual versus recent activity patterns. In addition, due to the limited granularity of the collected data, participants could not be classified into light, moderate, or vigorous activity groups to examine potential differences in model structure. Future research could employ more comprehensive physical activity measures to capture multiple dimensions and allow finer-grained analyses, which may provide deeper insights into the relationships between physical activity and phone addiction.

## 5 Conclusion

This study first examined the direct and indirect associations between physical activity and phone addiction among Chinese university students through the chain mediating role of resilience and subjective wellbeing. The indirect association between physical activity and phone addiction was mediated by the subjective wellbeing and the chain mediating role of resilience and subjective wellbeing. Nonetheless, further research is required to expand the results of this study by exploring the associations between physical activity and phone addiction in diverse populations.

## Data Availability

The raw data supporting the conclusions of this article will be made available by the authors, without undue reservation.
