# Supplementary material for: How physical activity protects against smartphone addiction: examining the mediating pathways of resilience and subjective wellbeing in Chinese university students
Source: Front Psychol. 2026 Jan 9;16:1704827. doi: 10.3389/fpsyg.2025.1704827 (PMC12827692; doi:10.3389/fpsyg.2025.1704827)
Supplement: Supplementary file 1 [file Data_Sheet_1.PDF]

Supplementary Materials

S1 Invariance testing results of the structural equation model across groups

Model	χ^2/DF	TLI	CFI	RMSEA	ΔCFI
Unconstrained	1.424	0.981	0.989	0.029	-
Measurement weights	1.336	0.985	0.99	0.026	0.001
Structural weights	1.569	0.974	0.981	0.033	-0.009
Structural covariances	1.599	0.973	0.979	0.034	-0.002
Structural residuals	1.642	0.971	0.976	0.035	-0.003
Measurement residuals	1.692	0.969	0.971	0.037	-0.005

Note. χ^2/DF = chi-square/degrees of freedom ratio; TLI = Tucker-Lewis Index; CFI = Comparative Fit Index; RMSEA = Root Mean Square Error of Approximation; ΔCFI = change in CFI relative to the previous, less constrained model. Values of CFI and TLI > 0.90 and RMSEA < 0.08 indicate acceptable fit; ΔCFI ≤ 0.01 suggests invariance across groups.
